# Exciton–polariton condensation in MAPbI_3_ films from bound states in the continuum metasurfaces

**DOI:** 10.1515/nanoph-2025-0128

**Published:** 2025-08-27

**Authors:** Marco Marangi, Andrea Zacheo, Alexander M. Dubrovkin, Giorgio Adamo, Cesare Soci

**Affiliations:** Division of Physics and Applied Physics, School of Physical and Mathematical Sciences, 54761 Nanyang Technological University, Singapore 637371, Singapore; Centre for Disruptive Photonic Technologies, 54761 TPI, Nanyang Technological University, Singapore 637371, Singapore; National Centre for Advanced Integrated Photonics (NCAIP), 54761 Nanyang Technological University, Singapore 639798, Singapore

**Keywords:** polariton condensate, halide perovskite film, dielectric metasurface, bound states in the continuum, integrated silicon photonics

## Abstract

Exciton–polariton condensation in optical resonators is a fascinating pathway to realise ultra-coherent states of light, underpinned by Bose–Einstein quasiparticles. Bearing inherently non-radiative nature, bound states in continuum (BIC) have proven to be an excellent platform to achieve polariton condensation. Here, we report exciton–polariton condensation in a solution-processed perovskite thin film hybridized with a silicon BIC metasurface. Thanks to the high quality factor of the BIC, the polariton condensation exhibits low fluence threshold, narrow linewidth, and large spatial and temporal coherence. These results demonstrate the feasibility of integration of perovskite polaritonic devices in scalable silicon photonic platforms.

## 1 Introduction

In 1929, von Neumann and Wigner demonstrated that Schrödinger’s equation can support solutions that are bound states lying in the continuum [1]. Initially regarded as a mathematical curiosity, BICs gained more interest when Herrick and Stillinger proposed that BICs could be found in semiconductor superlattices [2], [3]. Within this framework, Federico Capasso’s 1992 experimental realization of a semiconductor heterostructure superlattice with a positive-energy defect state with energy in the bandgap [4], [5], [6], ignited interest in the experimental implementations of BICs. While BICs can emerge in any wave-like system, photonics is arguably the field where they are making the most significant impact. BIC in photonic systems were formally predicted in 2008 – earlier theoretical predictions and experimental demonstrations exist which do not explicitly associate non-radiating modes to BICs – and since then symmetry protected and tunable BICs originating from interfering resonances, also called Friedrich–Wintgen BICs [7], have been observed in various optical cavities such as metasurfaces [8], [9], photonic crystals [10], distributed Bragg reflector, and waveguides [11].

Since photonic structures supporting BICs are intrinsically high-quality resonators – with ideally infinite *Q*-factor – they are excellent platforms to realize filters [12], sensors [13], lasers [14], [15], [16], [17], [18], and to induce strong light–matter interactions [19], [20], [21]. For the latter, the optical resonators must be integrated with strong excitonic materials. Halide perovskites, a family of hybrid organic–inorganic compound semiconductors with strong exciton binding energy and high luminescence yield have been recently employed to access the strong-coupling regime and form exciton polariton quasiparticles (formed by the hybridization of photons and excitons), up to room temperature [22], [23], [24], [25]. However, the realization of polariton condensates, a macroscopic state of matter in which the exciton polaritons behave coherently, has been mainly shown in high-quality perovskite crystals [19], [26], [27]. Recently, polariton condensation has also been achieved in polycrystalline films [28], [29] with much greater relevance toward scalability and optoelectronic integration. In this respect, methylammonium lead iodide (MAPbI_3_) films are particularly interesting due to their bright electroluminescence owing to good charge mobility and efficient charge injection, and have been successfully used for the realization of electrically-driven, light-emitting metadevices [30], [31].

In this work, we demonstrate condensation of exciton–polaritons in a solution-processed MAPbI_3_ film coupled to a silicon metasurface. The geometrical parameters of the nanopillars constituting the metasurface were carefully adjusted to match the high-Q BIC resonances with the MAPbI_3_ exciton energy, leading to band anti-crossing and the formation of upper and lower polariton bands. Thanks to the high-Q BIC, we observe exciton–polariton condensation and coherent radiation near the normal direction above a low excitation threshold of *P* = *P*_th_ ≅ 10.45 μJ cm^−2^. This is supported by the emergence of the characteristic hallmarks of polariton condensation, namely the strongly non-linear light–light curve, the reduction of emission linewidth, the blue shift of peak emission energy, and the increase of temporal and spatial coherence. These results mark a key demonstration of metasurface-assisted polariton condensation in a MAPbI_3_ polycrystalline film, offering a highly scalable and flexible solution for interfacing and integrating polariton condensate technologies with silicon photonics.

## 2 Main

**Figure 1:**
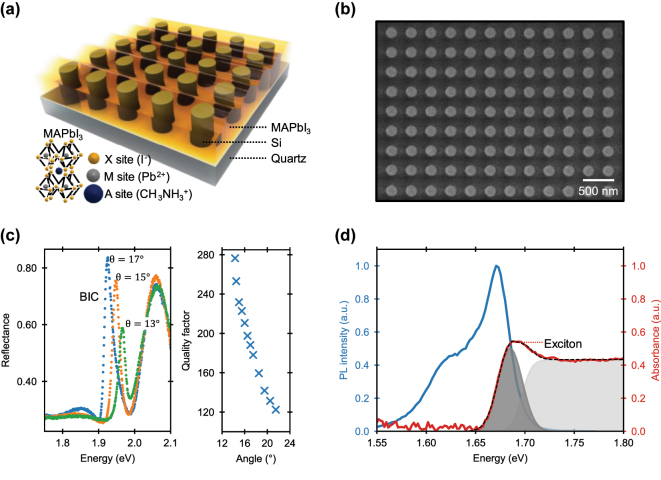
MAPbI_3_ perovskite film hybridized with a silicon bound state in the continuum (BIC) metasurface. (a) Schematic representation of a Si nanopillars BIC metasurface on a quartz substrate, covered by a polycrystalline MAPbI_3_ thin film. (b) Scanning electron microscopy (SEM) image of the Si BIC metasurface fabricated by electron beam lithography. (c) Left panel: reflectance spectra of the Si metasurface, with sharp BIC resonances which red-shift moving away from the normal direction of incidence (increasing *θ*); right panel: quality factor of the BIC resonance as function of incident angle, showing a steep increase while approaching the normal direction of incidence. (d) Photoluminescence (blue) and absorbance (red) spectra of the MAPbI_3_ perovskite film at 83 K. The black dashed line is the fit of the absorbance spectrum that includes the contributions from the exciton (dark grey shaded area) and the continuum (light grey shaded area) states, using the Elliott’s model.

In our study, we utilized 100 μm^2^ amorphous silicon metasurfaces coated by a polycrystalline MAPbI_3_ perovskite film, as illustrated by the schematic in Figure 1a, capped by a ∼1.7 μm thick layer of PMMA which serves as physical passivation [30]. The Si metasurfaces were fabricated via electron beam lithography (EBL) and dry etching (see Section 4) to obtain periodic arrays of nanopillars, as shown in the scanning electron microscope (SEM) image in Figure 1b. Accurate engineering of the Si nanopillars parameters allowed the formation of sharp BIC resonance. The 2-fold symmetry-protected BIC resonance [8], [32], [33] arises from an out-of-plane magnetic dipole (MD), generated by displacement currents around the normal direction (electric field distributions are shown in Figure S1). Away from the normal direction, the bound states become leaky with finite quality factors, appearing as a peak in reflectance (Figure 1c, left panel) with strong angular dispersion of the resonant energy and *Q* factor (Figure 1c, right panel). The MAPbI_3_ solution was cast on the Si metasurfaces via spin-coating, to form a polycrystalline film of 130 nm thickness, which showed high photoluminescence emission, peaked at ∼1.667 eV, and a strong excitonic line appearing as a peak in absorption ∼1.685 eV, when measured at 83 K (Figure 1d).

**Figure 2:**
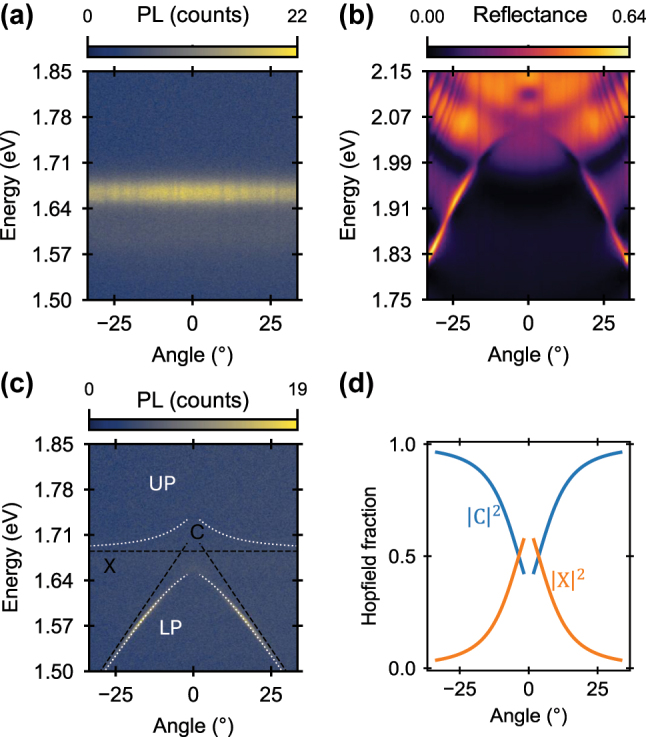
Strong coupling of perovskite film and Si-BIC metasurface at 83 K. (a) Experimental angle-resolved photoluminescence of the MAPbI_3_ film, showing isotropic excitonic emission at 1.667 eV. (b) Experimental angle-resolved TE-polarized reflectance of the metasurface, capped with a PMMA layer, showing a typical BIC resonance dispersion, strongly blue-shifted with respect to the MAPbI_3_ exciton energy, to account for the perovskite refractive index. (c) Experimental angle-resolved photoluminescence of the hybrid metasurface covered by the MAPbI_3_ film, displaying a distinct emission from the lower polariton branch. The black dashed lines indicate the dispersion of the exciton (*X*) and uncoupled BIC (*C*). The white dotted lines represent the calculated upper (UP) and lower (LP) exciton–polariton bands. (d) Calculated Hopfield coefficients of the experimental lower polariton, demonstrating high excitonic fraction ( ${\left|X\right|}^{2}$ ) and low photonic fraction ( ${\left|C\right|}^{2}$ ) around the Γ point, *θ* = 0°.

For strong coupling to take place, the decay rate of the excitonic and photonic components must be lower than the rate of energy exchange between the two [25]. The large oscillator strength of MAPbI_3_ and the high *Q* of dielectric metasurface are therefore essential conditions to observe strong light–matter coupling and the formation of exciton–polariton bands. The photoluminescence emission of the MAPbI_3_ film (at 83 K) is isotropic and peaks at 1.667 eV, as shown in the angle-resolved map of Figure 2a. The metasurface was designed to have a BIC resonance significantly blue shifted with respect to the MAPbI_3_ exciton, to account for the red-shift induced by the perovskite film. The experimental transverse electric (TE) polarization reflectance of the metasurface coated with PMMA (Figure 2b) shows the typical BIC dispersion with vanishing linewidth and increasing *Q* factor as it approaches the *θ* = 0, at ∼2.05 eV. To characterize the coupling of the photonic mode of the metasurface with the exciton of the perovskite, we start by measuring the angle-resolved photoluminescence (map shown in Figure 2c). The band of the coupled system is significantly red-shifted and has a different dispersion from that of the bare metasurface (Figure 2b), suggesting the formation of exciton–polaritons. To confirm that the system is in strong coupling, we fit the bands using the coupled oscillator model [10] between the MAPbI_3_ exciton and the photonic mode. The dispersion of the uncoupled photonic mode is linearly extrapolated from the lower polariton band at energies where the exciton contribution is negligible, following the procedure outlined in references [29], [34]. The coupled oscillator model captures well the experimental band dispersion (white dotted line in Figure 2c) and confirms the formation of exciton–polariton bands with a Rabi splitting of *ℏ*Ω = 84 meV (see Table S1 for the coupled oscillator model parameters). The lower polariton has a large excitonic component around normal incidence, as denoted by the Hopfield fraction (Figure 2d). This implies the presence of efficient relaxation pathways to the LP ground state, a condition which favours exciton–polariton condensation with low fluence threshold [35].

**Figure 3:**
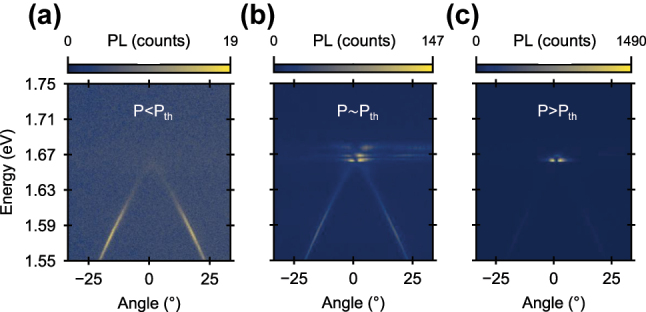
Exciton–polariton BIC condensation. Angle-resolved photoluminescence spectra, under non-resonant (*λ* = 400 nm) femtosecond pulsed excitation, illustrating the appearance of an exciton–polariton condensate around the BIC Γ point, with the increase of the pump fluence: (a) below the condensation threshold, *P* ∼ 2 μJ cm^−2^, (b) at the condensation threshold, *P* ∼ *P*_th_ = 10.45 μJ cm^−2^, and (c) above the condensation threshold, *P* ∼ 20 μJ cm^−2^.

The angle-resolved photoluminescence measurements show that the lower polariton state can be very efficiently populated, using low fluence ( $∼2\;\mu \text{J}\;\text{c}{\text{m}}^{-2}$ ) femtosecond laser excitation, as seen in Figure 3a. By increasing the excitation fluence to *P* ∼ *P*_th_ ≅ 10 μJ cm^−2^, we observe that the photoluminescence concentrates around *θ* = 0° and above the LP BIC band, which suggests the presence of non-linearity in the emission process and onset of polariton condensation (Figure 3b). For *P* > *P*_th_, the PL spectrum is dominated by two bright lobes around the Γ point, indicating emission with high directionality and narrow linewidth (Figure 3c), originating from the BIC state.

**Figure 4:**
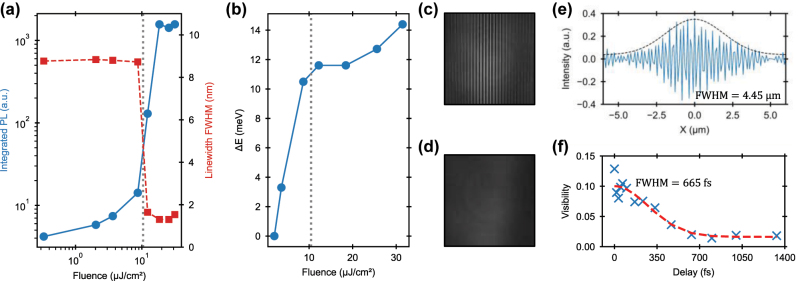
Coherent emission of the BIC polariton condensate. (a) Integrated photoluminescence emission intensity (blue) and linewidth (red) as function of excitation fluence, with the typical sigmoidal shape of the intensity and the sudden collapse of the linewidth at the fluence threshold of *P*_th_ = 10.45 μJ cm^−2^ (gray dotted line), indicating the transition from spontaneous emission to polariton condensate regime. (b) Blue-shift of the peak energy of the BIC polariton condensate as a function of fluence, typical of scattering phenomena in the polariton condensate. (c) and (d) Show the real space image, recorded on a CCD array, of the spatially superimposed emission of two arms in the Michelson interferometer, respectively after and before being temporally overlapped, with the typical interference fringes associated to coherence appearing at zero delay. (e) Intensity of the Fourier transform (FT) of a line cut of the real space image. The spatial coherence of the polariton condensate is extracted from the FWHM of the Gaussian function fitting of the envelope of the FT (black dashed line). (f) Fringe visibility as a function of delay time. The temporal coherence of the exciton–polariton condensate is extracted from the FWHM of the Gaussian fitting (red dashed line).

The integrated photoluminescence emission, as a function of excitation fluence, has the characteristic sigmoidal shape of polariton condensation (blue light–light curve in Figure 4a). The photoluminescence intensity first grows linearly until the fluence reaches the threshold *P*_th_, and then shows superlinear growth accompanied by a sudden linewidth reduction (red curve in Figure 4a), which signals an increase in temporal coherence. The observed fluence dependent blueshift of the peak energy of the LP BIC emission (Figure 4b) is a hallmark signature of polariton condensation, associated to polariton–polariton repulsive interactions. We measure the temporal and spatial coherence of the polariton condensate by sending the emitted light through a Michelson interferometer [36], [37], [38], where a beam splitter separates it into two arms with tunable distance. Before being recombined on a CCD camera, the wavefront of the beam in one of the two arms is flipped along one axis, using a retroreflector. By tuning the path difference, Δ*x*, between the two arms of the interferometer, it is possible to observe the formation of distinct interference fringes at the position of maximum overlap between the two beams (Δ*x* = 0 μm, Figure 4c), and determine the distance at which the coherence is lost and the fringes disappear (Δ*x* = 417 μm, Figure 4d). A spatial coherence of 4.45 μm was estimated by Gaussian fitting of the Fourier transform of the interference fringes at Δ*x* = 0 (Figure 4e). A temporal coherence of 665 fs was evaluated by measuring the visibility of the fringes as function of the temporal delay between the two arms of the interferometer. Despite the inhomogeneity of the MAPbI_3_ polycrystalline film, with sub-μm domains (Figure S6b), the spatial coherence length is comparable with those observed with single crystal perovskites [19], [27], [39], [40]. The low surface roughness of our films, of ∼5 nm (Figure S6a), is likely to benefit the onset of polariton condensation at low excitation fluence.

## 3 Conclusions

We reported the first demonstration of exciton–polariton condensation in polycrystalline MAPbI_3_ perovskite films. We confirmed the nature of the exciton–polariton condensate by measuring the characteristic exciton–polariton blueshift resulting from polariton–polariton interactions. Despite the inhomogeneity of the polycrystalline perovskite film we observed large spatial and temporal coherence of the polariton condensate emission. This was achieved by hybridizing the perovskite film with a silicon metasurface that supports a high quality factor BIC resonance. Building upon the seminal contributions Professor Federico Capasso gave to lasers, metasurfaces and flat optics, our work offers a promising route toward the development of large-area polaritonic and photonic laser sources, based on silicon platforms.

## 4 Methods

### 4.1 Metasurface design and fabrication

The silicon metasurfaces were designed via commercial software Ansys Lumerical using the rigorous coupled-wave analysis (RCWA) suite. The simulated nanopillars are 120 nm thick silicon with a radius of 71 nm and a unit cell period of 290 nm. The photonic bandstructure is calculated in the transverse electric (TE) mode.

Experimentally, BIC metasurfaces have been fabricated using e-beam lithography patterning and plasma etching of silicon films on quartz. Prior to the fabrication, quartz wafers have been sequentially sonicated in organic solvents (AR 600-71 remover (Allresist), acetone and IPA), followed by drying with nitrogen and short oxygen plasma treatment. Plasma enhanced chemical vapor deposition system (Cello Aegis-20) has been used to grow an amorphous Si layer at 300 °C. AR-N 7520 negative resist and Electra 92 conductive coating (Allresist) have been utilized for the lithography patterning (FEI Helios Nanolab), and SF6/O2 gas mixture for the plasma etching (Oxford PlasmaPro100 ICP-RIE). The resist removal has been performed using sequential treatment with AR 300-76 (Allresist), AR 600-71, acetone and IPA. Finally, the samples were dried with nitrogen and underwent short oxygen plasma treatment.

### 4.2 MAPbI_3_ perovskite synthesis and deposition

MAPbI_3_ films were fabricated from a 0.5 M precursor solution of CH_3_NH_3_I (Dyesol, Greatcell) and PbI_2_ (99.99 %, Sigma-Aldrich) with a molar ratio 1:1, in anhydrous dimethylformamide (DMF, Sigma-Aldrich). The solution was magnetically stirred over night at 373 K in N_2_ inert atmosphere glovebox and then filtered by a polyvinylidene fluoride (PVDF) syringe filter (0.45 μm) before spin-coating. Prior to deposition, the substrates were cleaned with ultrasonication for 5 min in acetone, isopropanol, and deionized water, respectively. Subsequently, substrates were dried with nitrogen, followed by 20 min ozone surface treatment. The perovskite precursor solution was spincoated onto the quartz substrates with a speed of 4,000 rpm for 35 s using the toluene anti-solvent deposition method, with the anti-solvent being drop-cast on the substrates 6 s after spinning began. The film was then annealed at 373 K for 15 min, yielding a 130 nm thickness, as measured by atomic force microscopy.

### 4.3 Angle-resolved spectroscopy

Angle-resolved photoluminescence (ARPL) measurements were performed with a custom built microspectrometer setup (schematic in Figure S5), consisting of an inverted optical microscope (Nikon Ti-u, 50× objective, NA = 0.55), a spectrograph (Andor SR-303i with a 300 lines/mm grating), and a charged-coupled detector (CCD, Andor iDus 420). A series of lenses along the beam path between the microscope and the spectrograph projects the back focal plane (BFP) of the collection objective on the slit of the spectrograph, allowing the collection of angular information within bounds defined by $\frac{k_{x}}{k_{0}}=\text{NA}=0.55$ . The sample was excited by a 400 nm fs pulsed laser with 1 KHz repetition rate.

### 4.4 Measurements of spatial and temporal coherence

The interference pattern is measured with a standard Michelson interferometer (schematic in Figure S5), where the sample emission is split into two arms by a 50/50 beam splitter. The two arms are mounted on translating stages to spatially and temporally overlap the reflect images with a retroreflector and a mirror to form interference fringes. The retroreflector is mounted on a coarse alignment stage, while the mirror is mounted on a precision micrometer actuator with 1 μm resolution (model SM1ZA). The two arms are then recombined on a CMOS camera with 3.45 μm × 3.45 μm pixel size (model CS165MU). A temporal delay is induced by translating one of the two arms to track the visibility as a function of delay time and thus define the temporal coherence of emission.

## Supplementary Material

Supplementary Material Details
