# Supplementary material for: Exciton–polariton condensation in MAPbI3 films from bound states in the continuum metasurfaces
Source: Nanophotonics. 2025 Aug 27;14(23):4241–7. doi: 10.1515/nanoph-2025-0128 (PMC12617696; doi:10.1515/nanoph-2025-0128)
Supplement: Supplementary file 1 — Supplementary Material Details [file j_nanoph-2025-0128_suppl_001.docx]

**Exciton-polariton condensation in MAPbI_3_ films from bound states in the continuum metasurfaces**

**Marco Marangi,^1,2^ Andrea Zacheo,^1,2^ Alexander M. Dubrovkin,^1,2^**

**Giorgio Adamo,^1,2^ Cesare Soci^1,2,3,*^**

*^1^Division of Physics and Applied Physics, School of Physical and Mathematical Sciences, Nanyang Technological University, Singapore 637371.*

*^2^Centre for Disruptive Photonic Technologies, TPI, Nanyang Technological University, Singapore 637371.*

*^3^National Centre for Advanced Integrated Photonics (NCAIP), Nanyang Technological University, Singapore 639798.*

[**csoci@ntu.edu.sg*](mailto:*csoci@ntu.edu.sg)

**Supplementary Material**

**
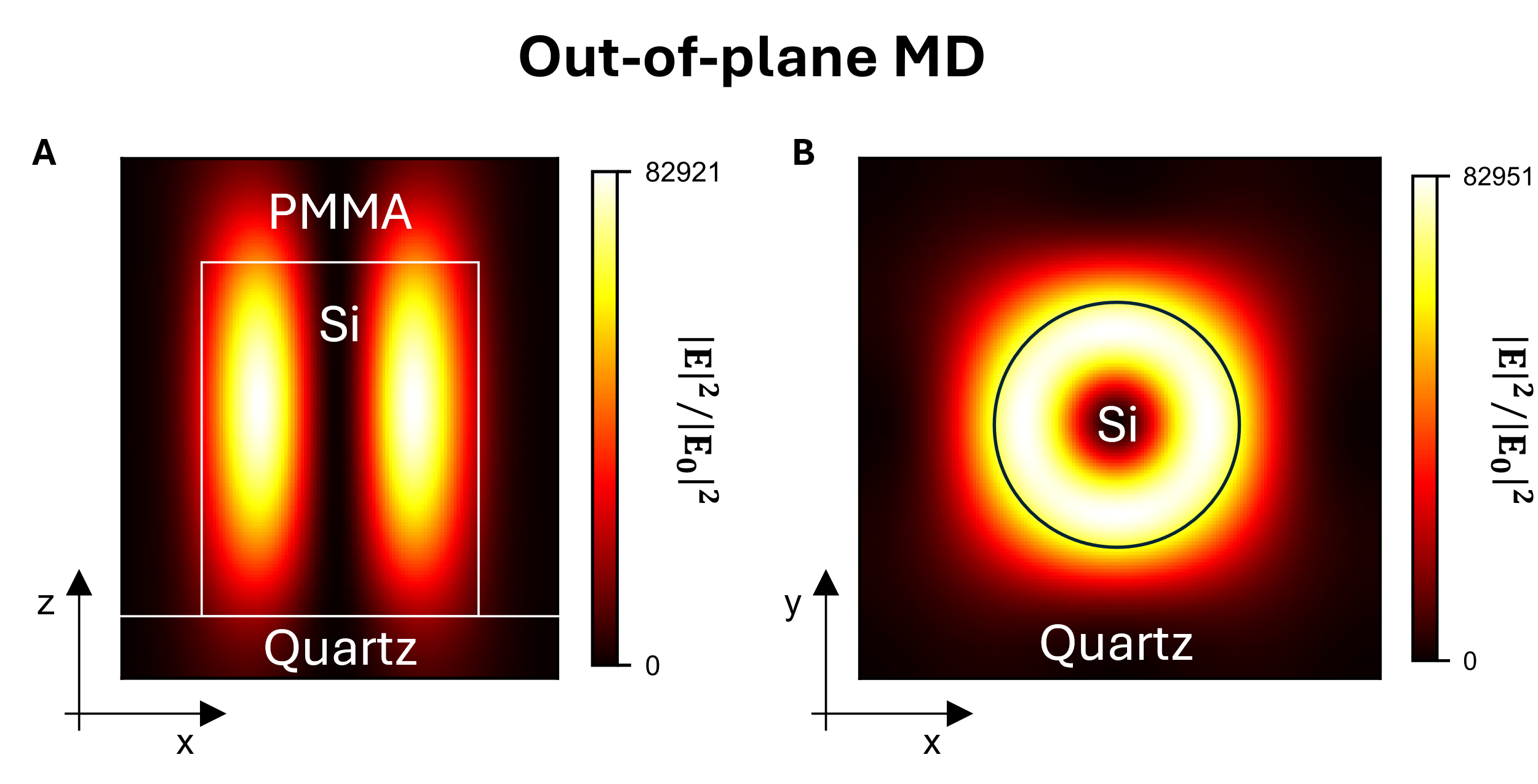
**

**Figure S1:** Simulated electric field distribution of the out-of-plane magnetic dipole BIC in the *xz* and *xy* plane (A and B, respectively).

**
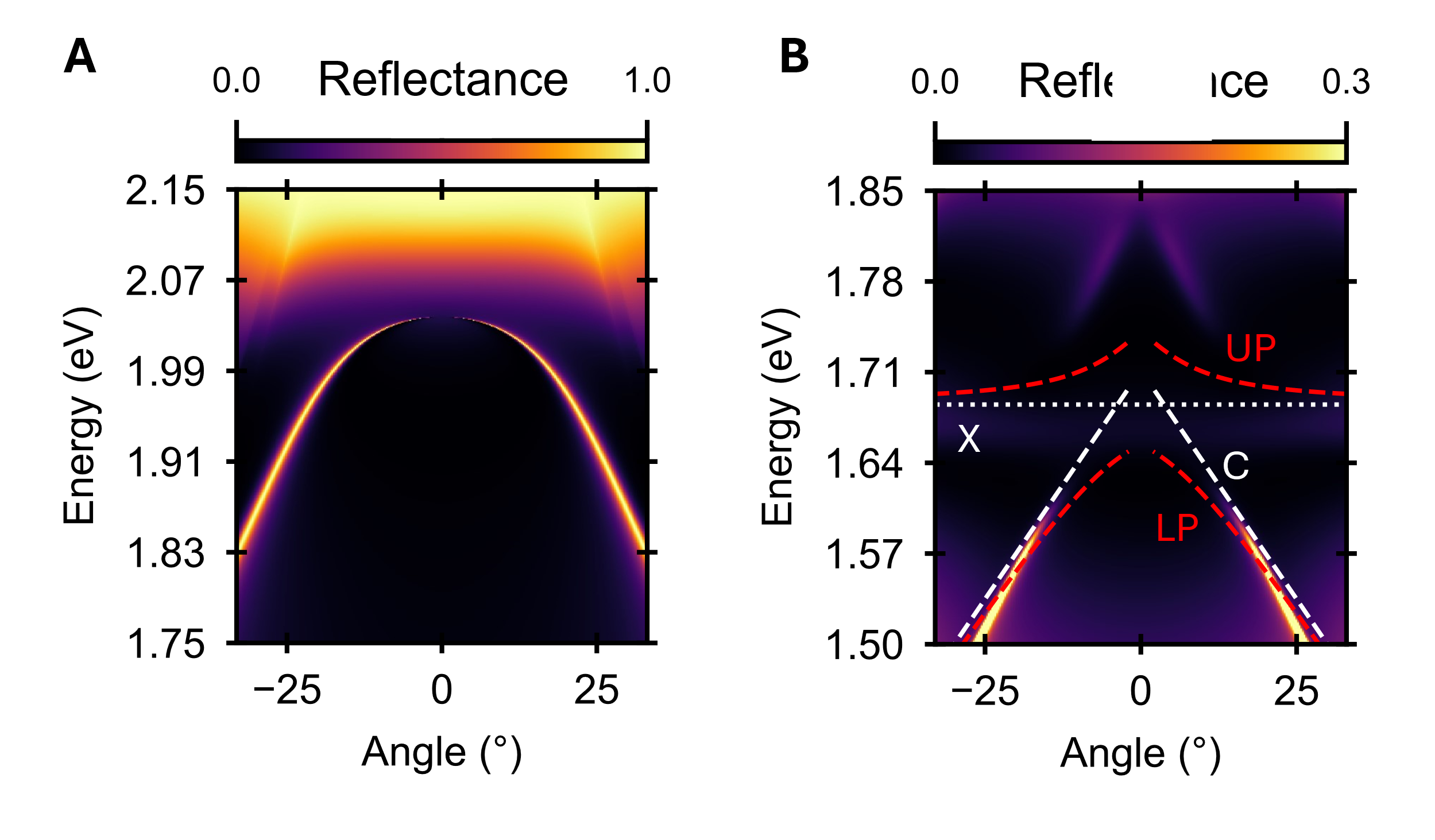
**

**Figure S2:** Simulated angle-resolved reflectance of the (**A**) bare and (**C**) active metasurface. The exciton-polariton dispersion in (**B**) correspond to the bands calculated with the coupled oscillator model in Figure 2C.


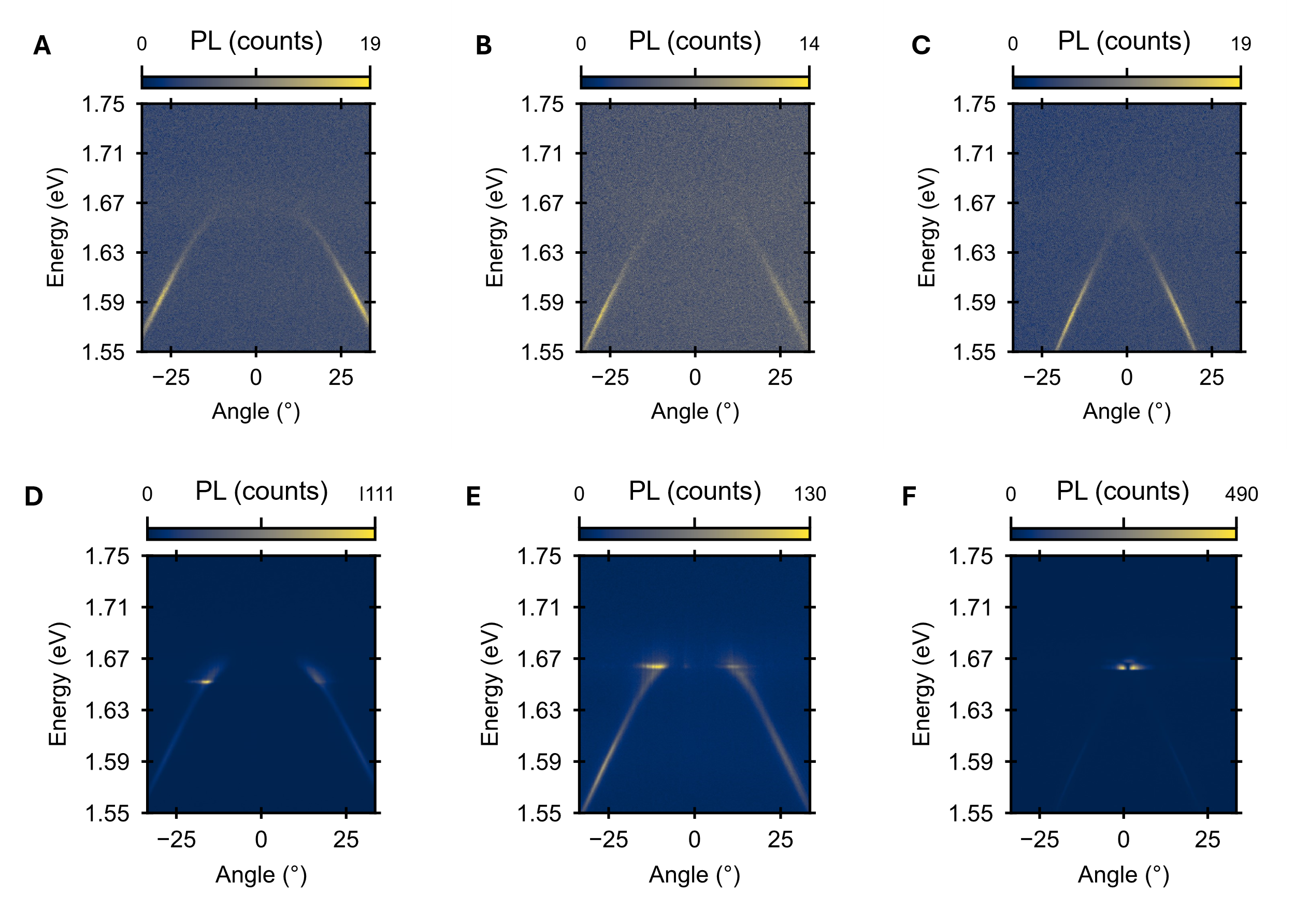
 **Figure S3:** Experimental angle-resolved photoluminescence of various strongly coupled BIC resonances with different cavity detunings (A,B,C) below and (D,E,F) above threshold. The polariton condensates showed in this figure occur in metasurfaces that are spatially separate in the range of hundreds of micrometers, highlighting the large-scale homogeneity of the hybrid structure.


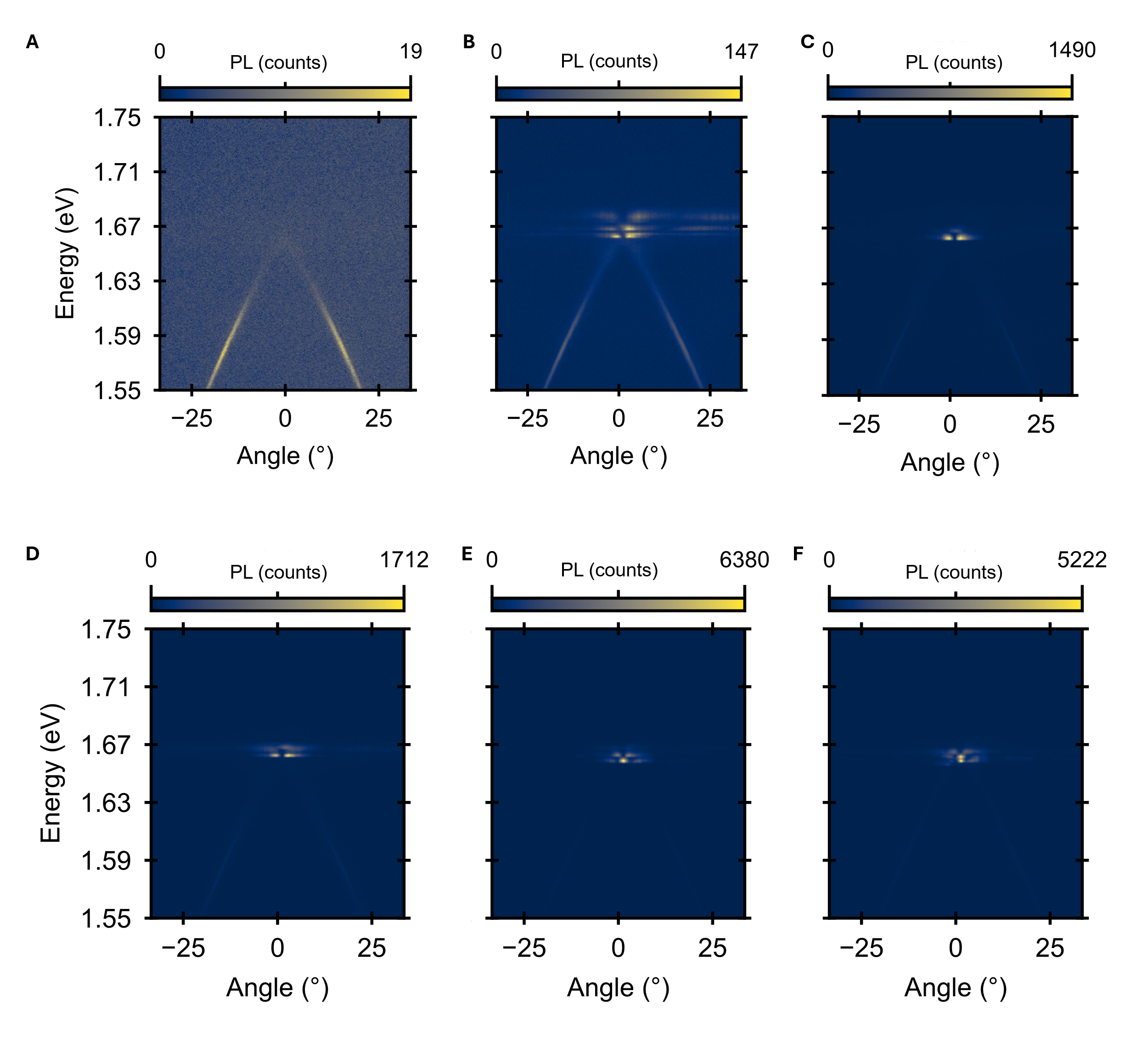


**Figure S4:** Experimental angle-resolved photoluminescence (A) below, (B) around, and (C-F) above threshold. At large fluences, a series of discretized modes of the polariton condensate arise due to polaritons interacting with local potentials within the pump spot as explained in [19], [20], [27].


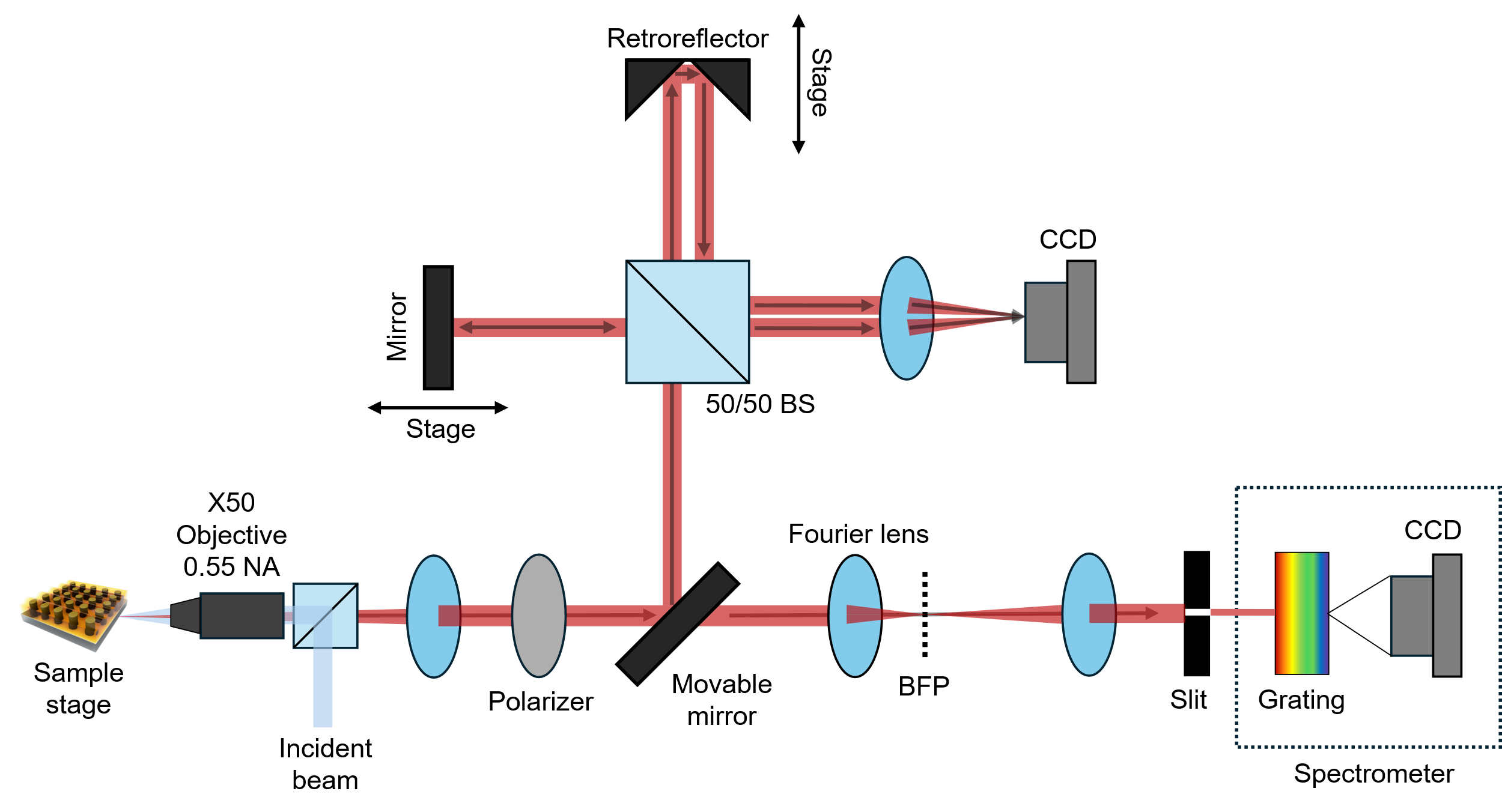


**Figure S5:** Schematic of the Fourier imaging setup used to measure the angle-resolved reflectance and photoluminescence bands of the metasurfaces. A Michelson interferometer is inserted into the collection path via a movable mirror to measure the temporal and spatial coherence of the polariton condensate.


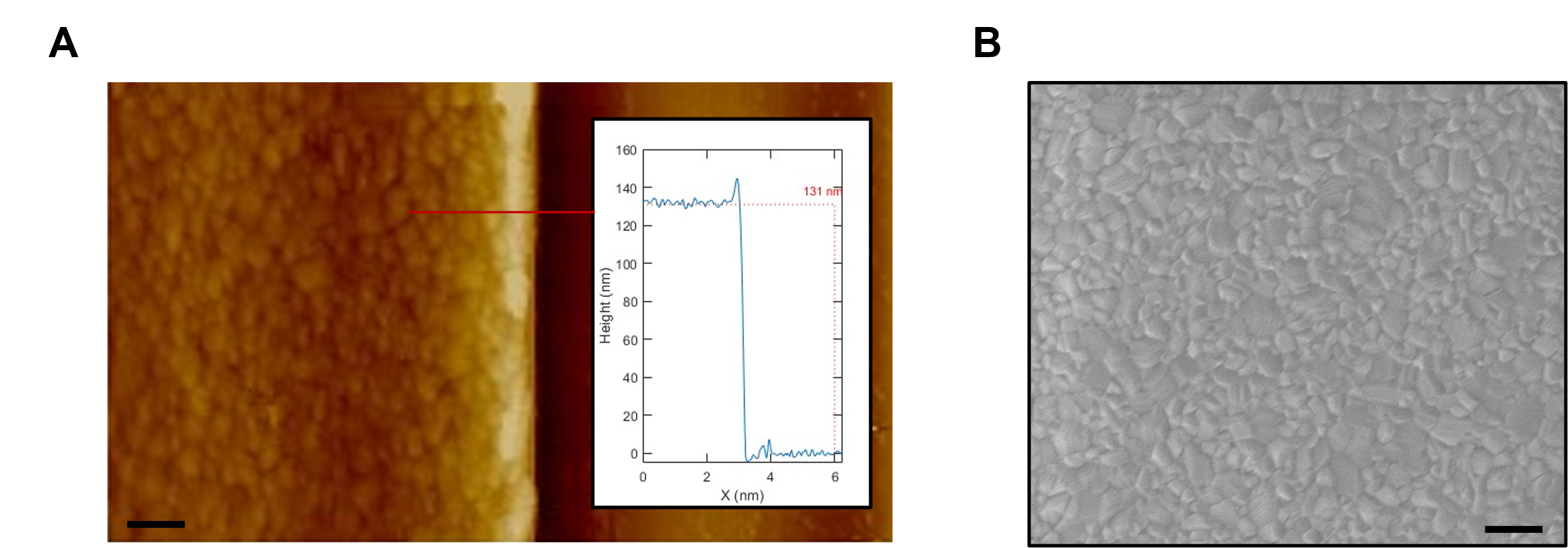


**Figure S6: A)** Atomic force microscopy area scan of the perovskite film resulting in an absolute roughness of 4.94 nm. The inset shows the line scan used to determine a film thickness of 130 nm. **B)** Scanning electron microscope image of the polycrystalline perovskite film showing sub-micron grain size. The scalebar is 500 nm in both panels.

**Table S1:** Fitting parameters of the coupled oscillator model corresponding to the exciton-polariton curves in Figure 2C.

|  | **Energy (eV)** | **Derivation** |
| --- | --- | --- |
| Exciton energy | 1.685 | From experimental absorption spectrum |
| Average exciton linewidth | 0.028 | From experimental absorption spectrum |
| Cavity resonance energy | 1.710 | Linear extrapolation of experimental lower polariton dispersion |
| Average cavity linewidth | 0.015 | Linear extrapolation of experimental lower polariton dispersion |
| Rabi splitting | 0.084 | Coupled oscillator model |
| Detuning | 0.025 | Linear extrapolation of experimental lower polariton dispersion |
